# Supplementary material for: Expanded Archaeal Genomes Shed New Light on the Evolution of Isoprenoid Biosynthesis
Source: Microorganisms. 2024 Mar 30;12(4):707. doi: 10.3390/microorganisms12040707 (PMC11052028; doi:10.3390/microorganisms12040707)
Supplement: Supplementary file 1 [file microorganisms-12-00707-s001.zip › Supplementary Figures.pdf]

Supplementary materials

# Expanded archaeal genomes shed new light on the evolution of archaeal isoprenoid biosynthesis and eukaryogenesis

Pengfei Zhu[1#], Jialin Hou[1#], Yixuan Xiong[1], Ruize Xie[1], Yinzhao Wang[2], Fengping Wang[1,2,3*]

1. School of Oceanography, Shanghai Jiao Tong University, Shanghai, China

2. State Key Laboratory of Microbial Metabolism, School of Life Sciences and Biotechnology, Shanghai Jiao Tong University, Shanghai, China.

3. Southern Marine Science and Engineering, Guangdong Laboratory (Zhuhai), Zhuhai, China.

[#]Pengfei Zhu and Jialin Hou contributed equally to this study.

*Correspondence: fengpingw@sjtu.edu.cn

# Catalogue

**Figure S1** The phylogenetic tree of the HMGCR-2 homologs. The maximum-likelihood tree was constructed using IQTREE2 with LG+R9+C60 model.

**Figure S2** The phylogenetic tree of the IDI-2 homologs. The maximum-likelihood tree was constructed using IQTREE2 with LG+R9+C60 model.

**Figure S3** The phylogenetic tree of the AMPD and PMD homologs. The maximum-likelihood trees were constructed using IQTREE2 with LG+R8+C60 and LG+R8+C60 model, respectively.

**Figure S4** The phylogenetic tree of the AcnX1 homologs. The maximum-likelihood tree was constructed using IQTREE2 with LG+R8+C60 model.

**Figure S5** Gene clusters encoding Eukaryote-type and Thermoplasma-type MVA pathway in the Asgard contigs

**Figure S6** The conserved motif sequences in PMD and DMD.

# S1

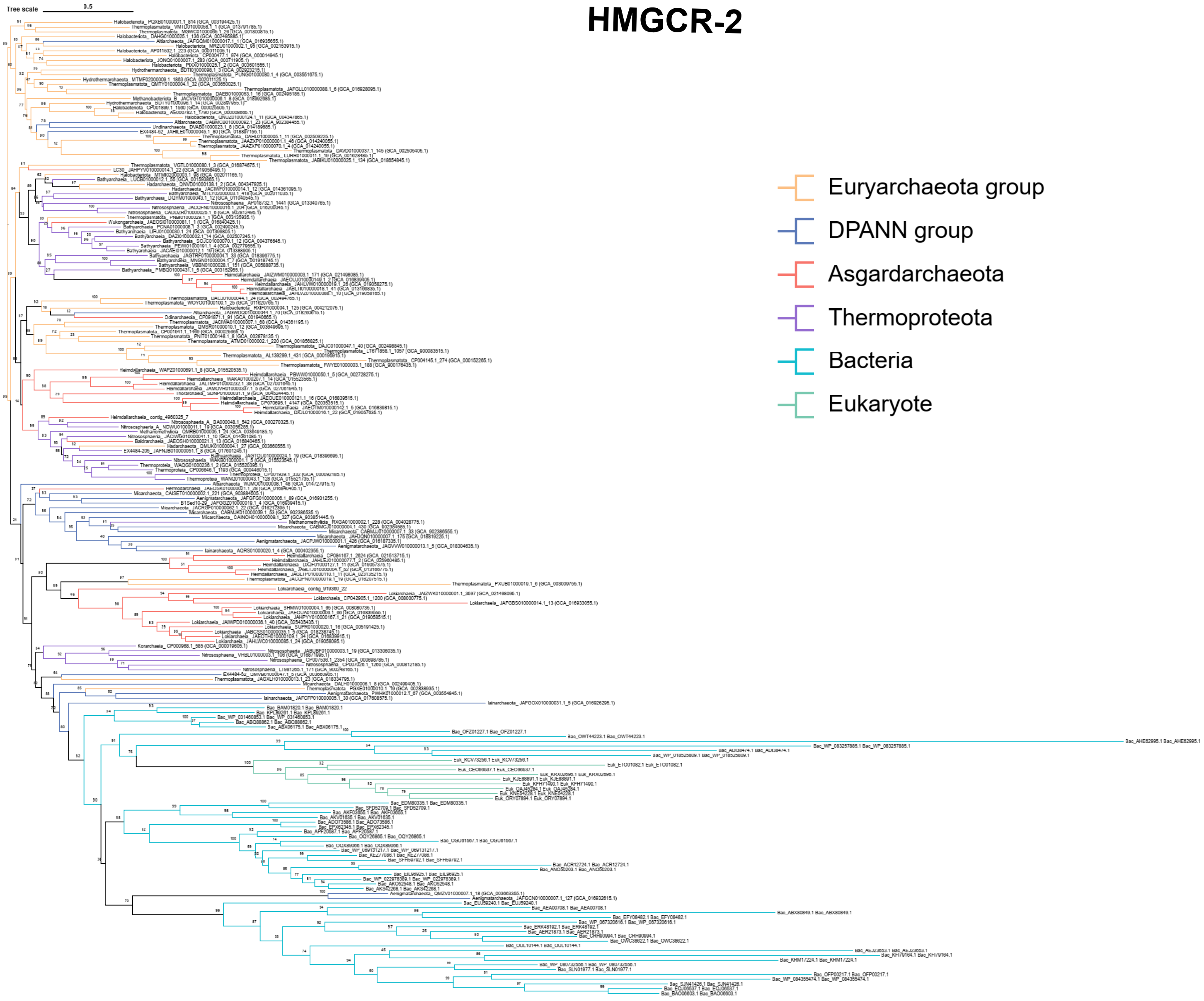
Tree scale 0.5
HMGCR-2
Euryarchaeota group
DPANN group
Asgardarchaeota
Thermoproteota
Bacteria
Eukaryote

# S2

## IDI-2

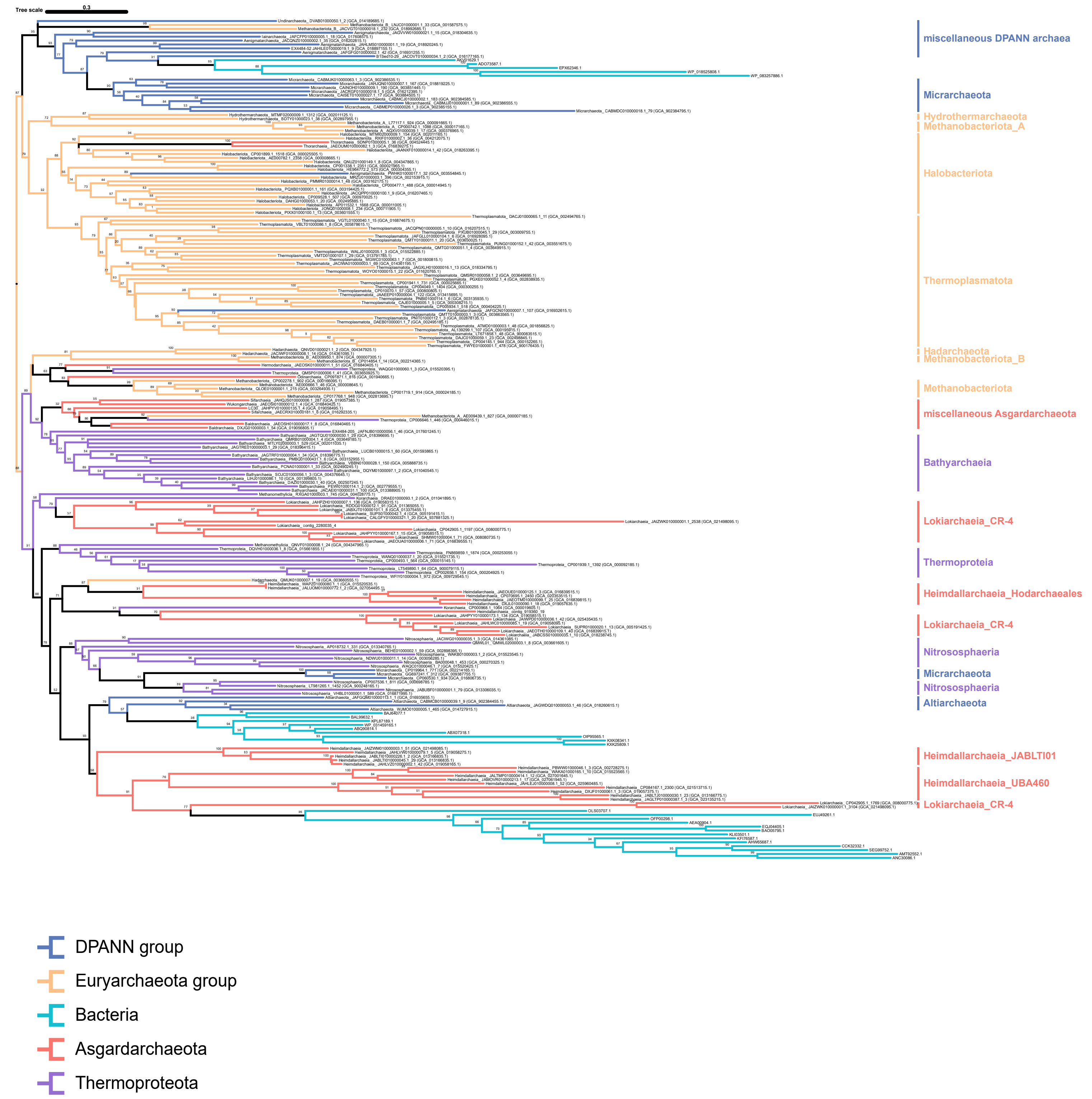
Tree scale
0.3
miscellaneous DPANN archaea
Micrarchaeota
Hydrothermarchaeota
Methanobacteriota_A
Halobacteriota
Thermoplasmatota
Hadarchaeota
Methanobacteriota_B
Methanobacteriota
miscellaneous Asgardarchaeota
Bathyarchaeia
Lokiarchaeia_CR-4
Thermoproteia
Heimdallarchaeia_Hodarchaeales
Lokiarchaeia_CR-4
Nitrososphaeria
Micrarchaeota
Nitrososphaeria
Altiarchaeota
Heimdallarchaeia_JABLTI01
Heimdallarchaeia_UBA460
Lokiarchaeia_CR-4
DPANN group
Euryarchaeota group
Bacteria
Asgardarchaeota
Thermoproteota

# S3

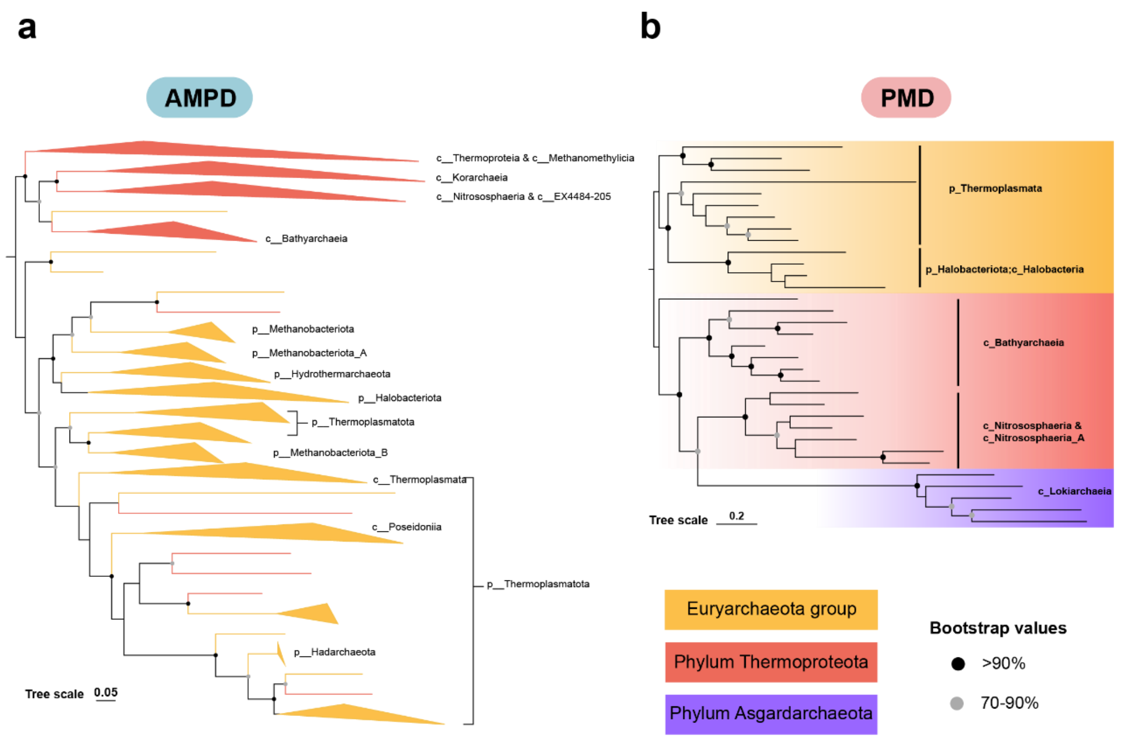
a
b
AMPD
c__Thermoproteia & c__Methanomethylicia
c__Korarchaeia
c__Nitrososphaeria & c__EX4484-205
c__Bathyarchaeia
p__Methanobacteriota
p__Methanobacteriota_A
p__Hydrothermarchaeota
p__Halobacteriota
p__Thermoplasmatota
p__Methanobacteriota_B
c__Thermoplasmata
c__Poseidoniia
p__Thermoplasmatota
p__Hadarchaeota
Tree scale 0.05
PMD
p_Thermoplasmata
p_Halobacteriota;c_Halobacteria
c_Bathyarchaeia
c_Nitrososphaeria &
c_Nitrososphaeria_A
c_Lokiarchaeia
Tree scale 0.2
Euryarchaeota group
Phylum Thermoproteota
Phylum Asgardarchaeota
Bootstrap values
>90%
70-90%

# S4

# AcnX1

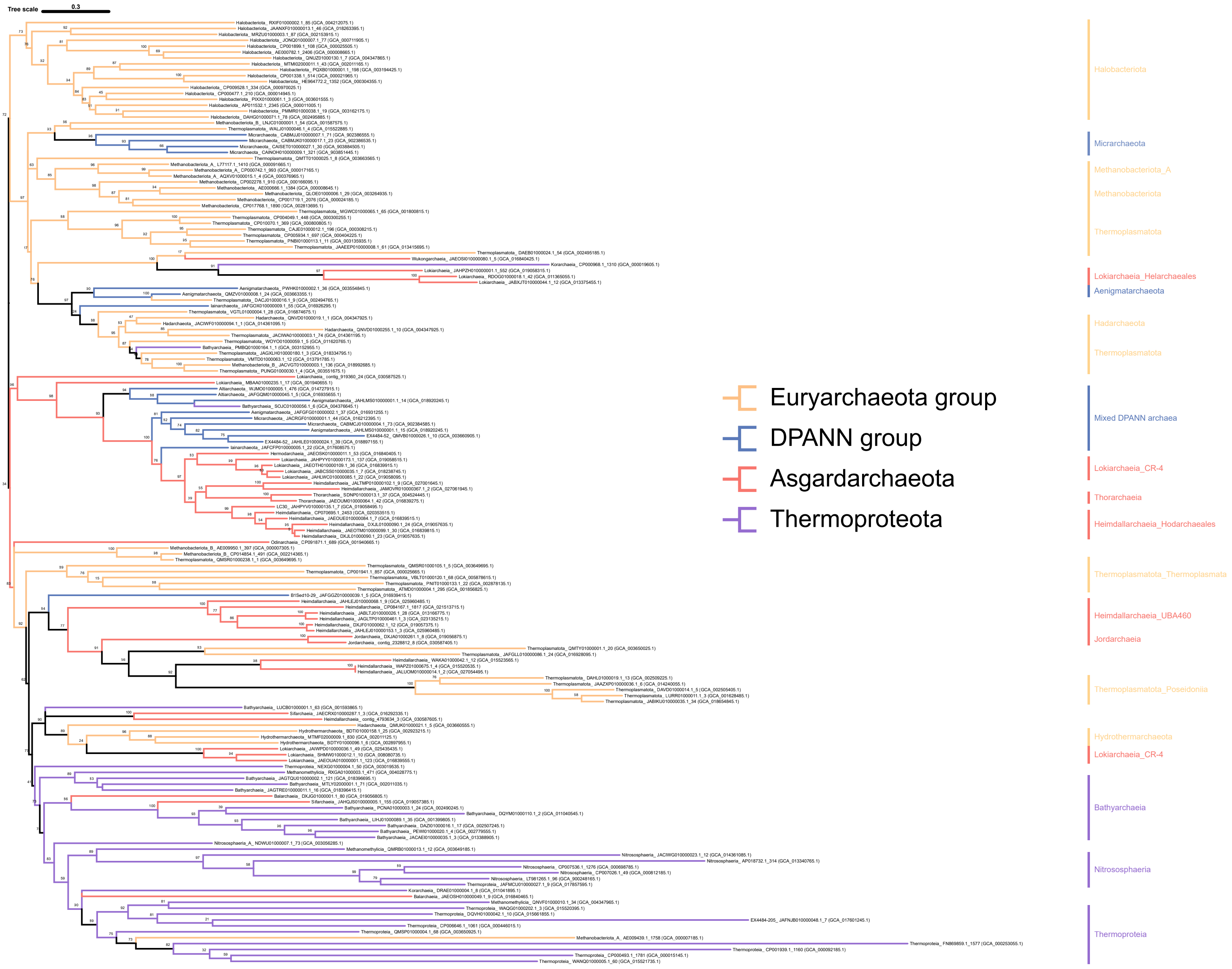

Tree scale
0.3
Euryarchaeota group
DPANN group
Asgardarchaeota
Thermoproteota
Halobacteriota
Micrarchaeota
Methanobacteriota_A
Methanobacteriota
Thermoplasmatota
Lokiarchaeia_Helarchaeales
Aenigmatarchaeota
Hadarchaeota
Thermoplasmatota
Mixed DPANN archaea
Lokiarchaeia_CR-4
Thorarchaeia
Heimdallarchaeia_Hodarchaeales
Thermoplasmatota_Thermoplasmata
Heimdallarchaeia_UBA460
Jordarchaeia
Thermoplasmatota_Poseidoniia
Hydrothermarchaeota
Lokiarchaeia_CR-4
Bathyarchaeia
Nitrososphaeria
Thermoproteia

# S5

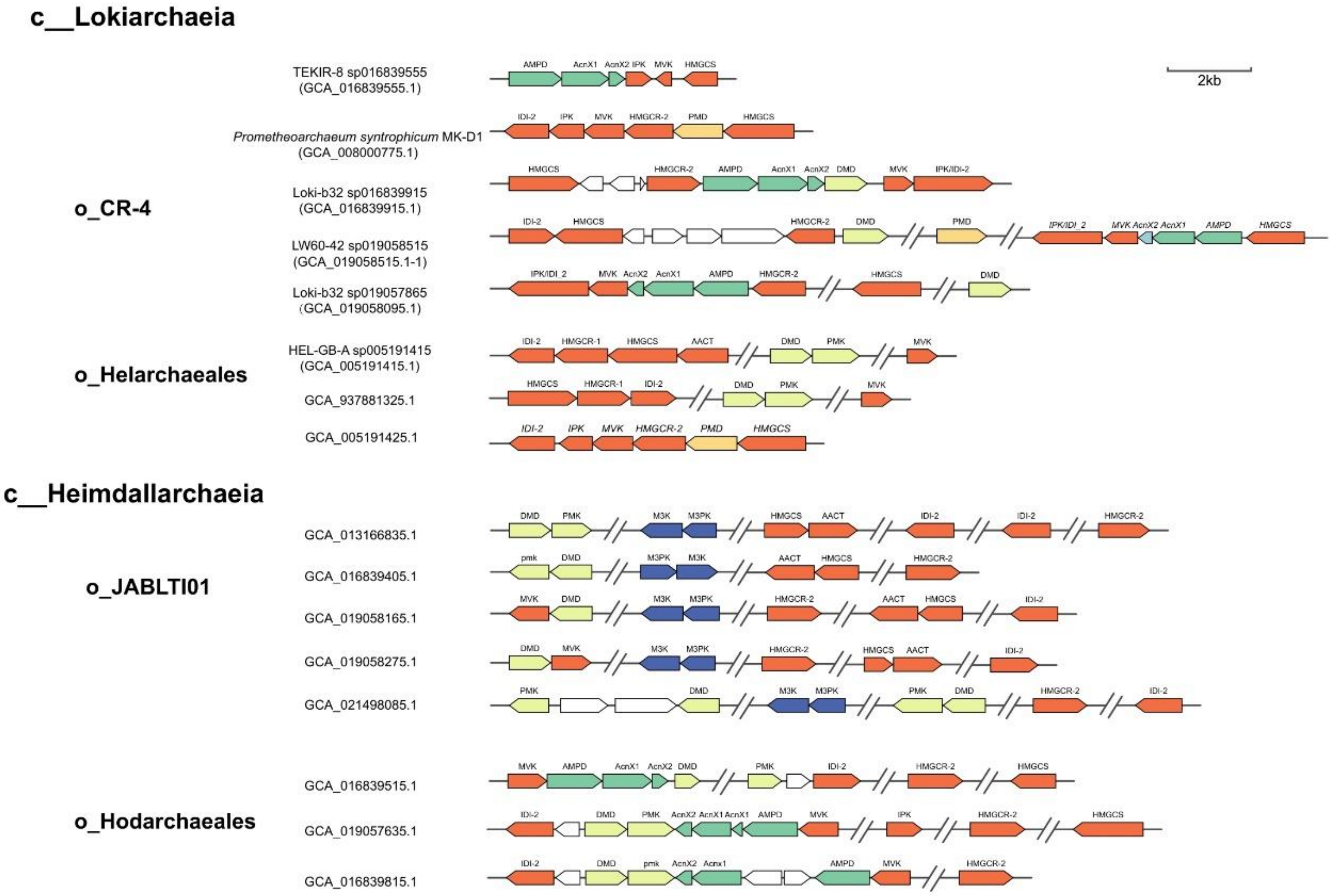
c__Lokiarchaeia
2kb
TEKIR-8 sp016839555
(GCA_016839555.1)
*Prometheoarchaeum syntrophicum* MK-D1
(GCA_008000775.1)
o_CR-4
Loki-b32 sp016839915
(GCA_016839915.1)
LW60-42 sp019058515
(GCA_019058515.1-1)
Loki-b32 sp019057865
(GCA_019058095.1)
o_Helarchaeales
HEL-GB-A sp005191415
(GCA_005191415.1)
GCA_937881325.1
GCA_005191425.1
c__Heimdallarchaeia
GCA_013166835.1
o_JABLTI01
GCA_016839405.1
GCA_019058165.1
GCA_019058275.1
GCA_021498085.1
GCA_016839515.1
o_Hodarchaeales
GCA_019057635.1
GCA_016839815.1

# S6

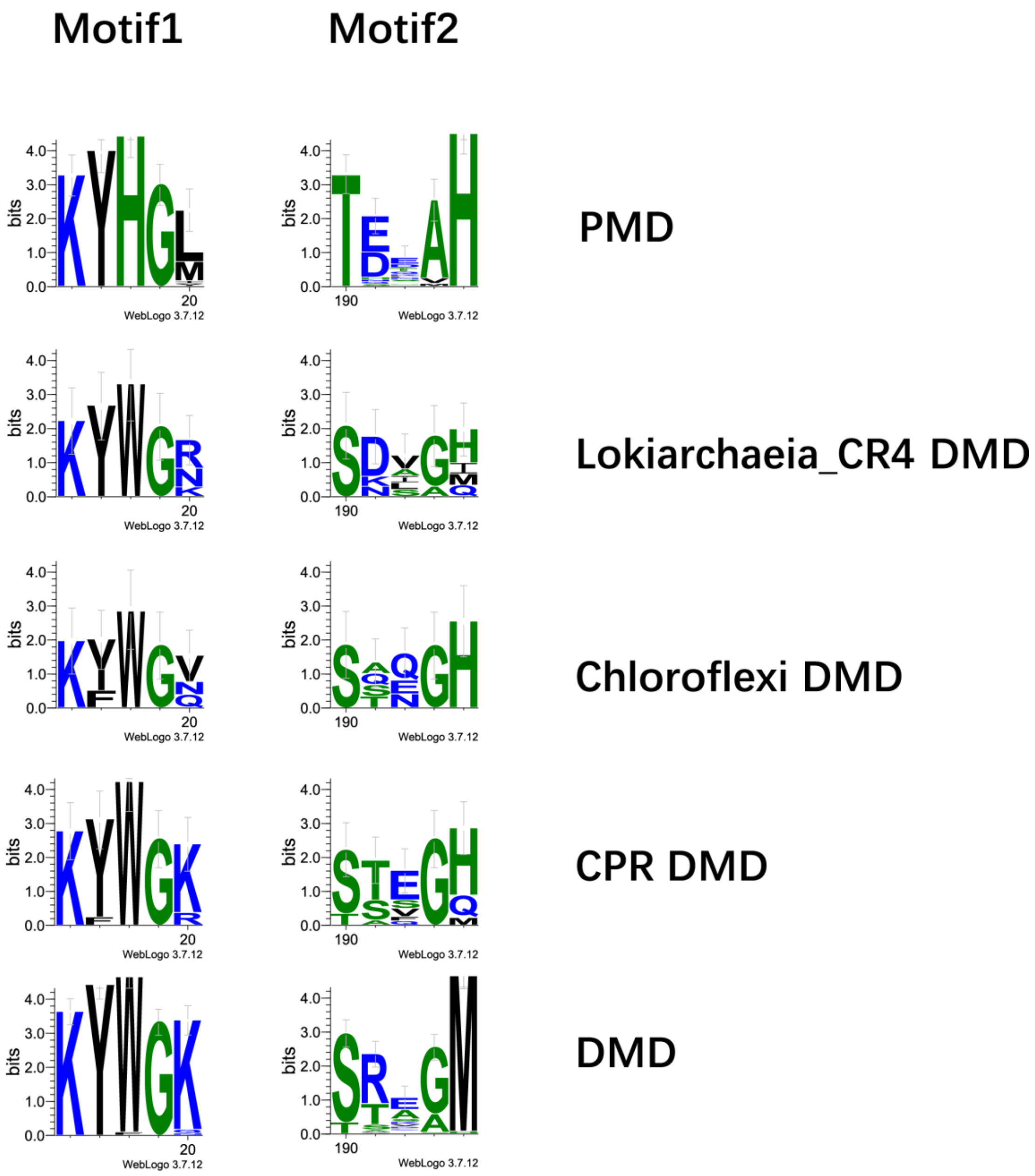
Motif1
Motif2
PMD
Lokiarchaeia_CR4 DMD
Chloroflexi DMD
CPR DMD
DMD
bits
WebLogo 3.7.12
